# Unicystic Ameloblastoma Mimicking Lateral Periodontal Cyst

**DOI:** 10.1155/2022/8197837

**Published:** 2022-02-14

**Authors:** Maya Fedhila, Raouaa Belkacem Chebil, Sonia Karray, Badreddine Sriha, Lamia Oualha, Nabiha Douki

**Affiliations:** ^1^Department of Oral Medicine and Oral Surgery, SAHLOUL Hospital (Sousse), Dental Faculty of Monastir, University of Monastir, Tunisia; ^2^Laboratory of Oral Health and Maxillofacial Rehabilitation (LR12ES11), University of Monastir, Tunisia; ^3^National Center of Medicine in Schools and Universities, Tunisia; ^4^Faculty of Medicine, University of Sousse, Farhat Hached Hospital of Sousse, Department of Pathology, Sousse, Tunisia

## Abstract

Intraosseous unicystic ameloblastoma (UA) is a rare subtype of a true neoplasm of odontogenic epithelial origin: ameloblastoma. Despite its rareness, dealing with UA is problematic. It is usually mistaken for an odontogenic cyst, and biopsy is rarely relevant because of its multiple growth patterns. The biggest challenge remains the treatment choice. When we are faced with a mural UA presenting strong similarities with a lateral periodontal cyst and having high rates of recurrence, how is the balance found between the young age, psychological fragility, postoperative process, and need for diagnostic biopsy? That was our dilemma. Our patient is a 23-year-old man with a mural unicystic ameloblastoma, diagnosed with general anxiety disorder. The final decision was to turn to a simple enucleation because of the small size of the lesion, and its radiological features strongly evoked a lateral periodontal cyst. Besides, his young age, psychological condition, and UA's proximity to the surrounding soft tissues guided us toward simple enucleation. Two years later, no sign of radiological recurrence was noted. However, we are aware of a later possibility of resection in case of recurrence.

## 1. Introduction

Odontogenic lesions are usually discovered through a routine radiography exam, although swelling may appear in the oral cavity. In the setting of this presentation, cysts (e.g., dentigerous cyst, radicular cyst, and keratocyst) are the primary suspected lesion. However, ameloblastoma remains a distinct possibility.

The latter lesion is a slow-growing though locally aggressive tumor representing 9% to 10% of odontogenic tumors. Its frequently reported location is the mandible (angle and ramus region), often associated with an unerupted third molar. It occurs generally during the 3rd to 4th decade of life and has an equal sex distribution [1, 2].

Ameloblastoma is a neoplasm of odontogenic epithelium, arising from epithelial cellular elements and dental tissues in their various phases of development [2]. In pathology, the lesion is characterized by its recapitulation of embryologic ameloblasts and stellate reticulum. It may grow to a great size, causing facial asymmetry, displacement of teeth, malocclusion, and even pathologic fracture [3].

According to the 4th Edition of the World Health Organization update, ameloblastoma is classified into four categories: conventional/multicystic, extraosseous/peripheral, metastasizing, and unicystic [4]. Conventional ameloblastoma is the most common variant (86%), also characterized by the highest recurrence rates [3]. On the contrary, unicystic ameloblastoma (UA) is less recurrent and not due to a secondary cystic change. It is a unique de novo neoplasm representing 15% of ameloblastoma cases [5].

Histologically, we can identify three subtypes of growth patterns [5]:

- Luminal showing a flat ameloblastic cyst lining
- Intraluminal characterized by a tumor growth into the cyst lumen, usually with soft luminal projections
- Mural exhibiting infiltrating growth into the wall of the cyst and even beyond into the surrounding bone

UA is particularly known for multiple growth patterns in the same lesion. Hence, a biopsy might give the wrong diagnosis when the specimen shows only one of lesion's multiple growth patterns. It is a dilemma since the treatment of one can promote the recurrence of the other [1].

Currently, conservative and radical treatments exist for UA. The conservative approach consists of a simple enucleation (i.e., surgical removal of the whole lesion including the capsular/pseudocapsular surface), or an enucleation followed by Carnoy's solution [6], or a marsupialization followed by enucleation [1, 7].

On the other hand, radical treatment consists of surgical resection (with or without continuity defect) of the lesion and surrounding bone [1]. The invasive approach is generally indicated for the mural subtype, whereas the conservative one usually suits the luminal or intraluminal pattern growth groups [8].

Therefore, we will introduce a case of a mural UA in the right mandible sector of a 23-year-old man presenting a general anxiety disorder.

## 2. Case Description

A 23-year-old male presented to the dental unit of Sahloul Hospital in July 2019 due to the reappearance of an old swelling in his right mandible two months prior. He reported a self-resolving swelling episode that occurred one year before that. The patient was also followed in the psychiatry department for generalized anxiety disorder. No history of drug allergy was known. Extraoral physical examination revealed a swelling in the middle of the right mandible body with no associated lymphadenopathy or mouth-opening restriction.

**Figure 1:**
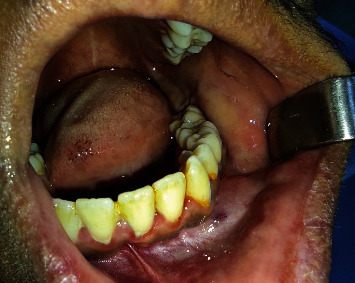
Aspect of the intraoral swelling.

On intraoral examination, the crown of tooth 35 was distally tipped, and a swelling was located in the low vestibule between teeth 35 and 36 (Figure 1). It extended to the jugal mucosa, measuring 0.7 cm by 0.5 cm. It was a nontender mass, hard in consistency, painful to palpation, and covered by normal oral mucosa. The cold test was positive for both 35 and 36, proving their vitality, and axial percussion test was negative for 35 and unsure concerning the first molar.

**Figure 2:**
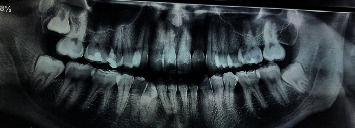
Panoramic radiograph from 2016 demonstrating a well-defined unilocular radiolucent lesion in the left mandible.

An orthopantomogram ordered in 2016 (Figure 2) revealed a well-circumscribed, unilocular radiolucent lesion with a sclerotic border between the second premolar and first molar roots, deforming and curving 35 root to the mesial side. The lesion seemed to arise from the lamina dura of 35, extending from the amelocemental junction to the apical third of the teeth along the periodontal space.

**Figure 3:**
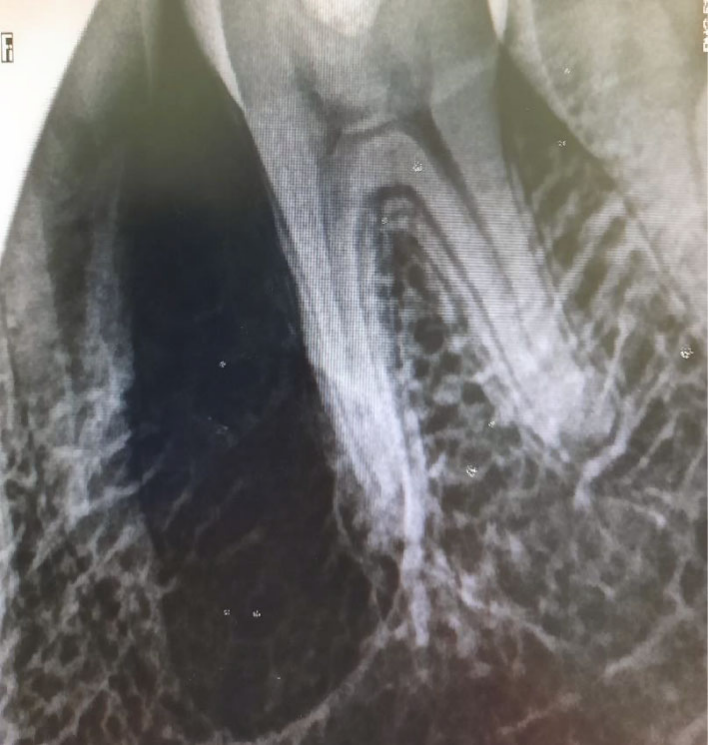
Radiovisiography taken the day of the consultation (2019).

On the day of the consultation, radiovisiography (Figure 3) was performed to determine the intraosseous progression of the lesion. A bigger image presenting the same features as that found in the panoramic radiography was depicted The lesion was then approximately 0.2 cm beyond the 35 root and 0.6 cm beyond the 36 distal root. The tooth sides in contact with the lesion (mesial side of the 35 root and mesial side of the 36 mesial root) were lacking periodontal space.

**Figure 4:**
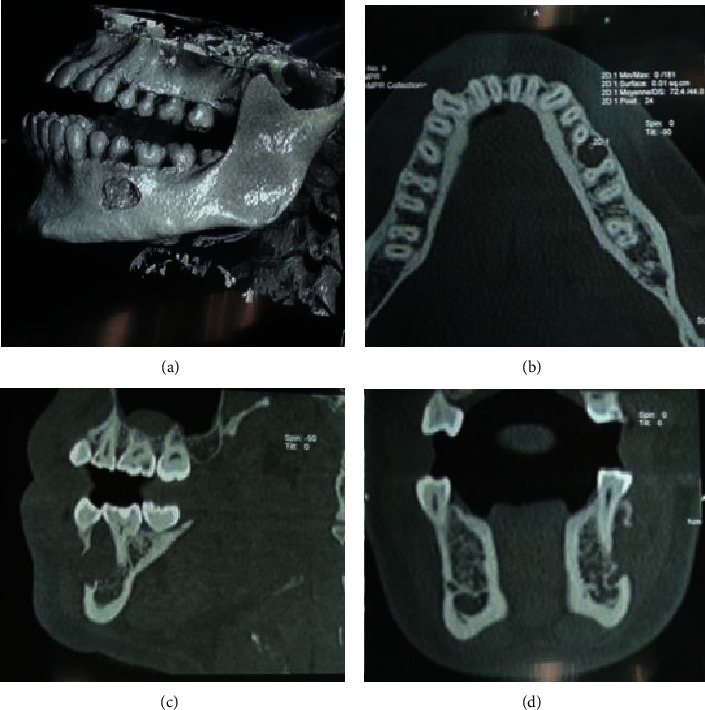
Dentascan radiography presenting the lesion on the left mandible on (a): a 3D reconstruction; (b): an axial view; (c): a sagittal view; and (d): a coronal view.

Computed tomography (CT) Dentascan was prescribed and showed a cystic lesion with a radiological tissue density confined in the mandible body (Figure 4). The buccal cortical bone was partially destroyed in its middle segment and thinned out in its upper portion. Deformation of the lingual cortical plate was observed due to the lesion expansion. The cortical bone of the mandibular canal was intact. No sign of root resorption was noted, and only signs of local aggressive behavior were observed.

Considering clinical and radiological findings, a lateral periodontal cyst was strongly suspected because of the lesion's location, its small size, the vitality of 35 and 36, the lesion's radiological continuity with the 35 lamina dura, and its slow evolution since the first radiograph. Giant cell granuloma and ameloblastoma were also thought of as possible differential diagnoses because of the local aggressive features noted in the CT Dentascan.

Because of the strong suspicion of the lateral periodontal cyst, the small size of the lesion, patient's young age, and his fragile psychological health, we opted for the enucleation of the lesion. We decided not to perform a biopsy since the lesion was too small. Besides, the entire specimen will be needed later to determine all the lesion's histological aspects especially in the case of ameloblastoma.

**Figure 5:**
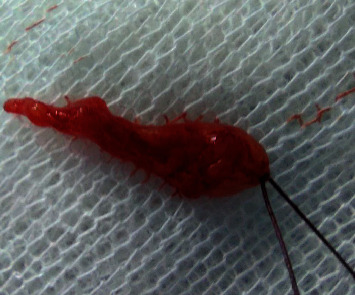
Resected specimen.

**Figure 6:**
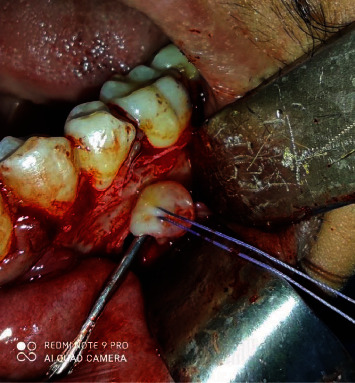
Enucleation of the lesion.

The patient was informed about the therapeutic options available and the risks of each one. The patient himself asked for the less-traumatic treatment since he was very agitated and could not handle substantial postoperative recovery. Therefore, under local anesthesia, the enucleation of the lesion was performed. The specimen (Figure 5) had a smooth surface. A cystic fluid was present on and around the lesion, appearing more clearly on aspiration. It was removed intact and sent for an anatomical pathology exam (Figure 6). Attention was paid not to make a vigorous curettage that might facilitate recurrence in the case of ameloblastoma. Both teeth around the lesion were left intact.

**Figure 7:**
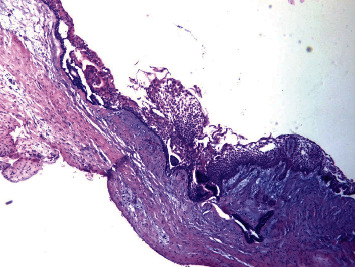
Optical microscopic picture presenting ameloblastomatous epithelium lining the cyst cavity. The specimen was stained with hematoxylin and eosin, magnified 40 times.

**Figure 8:**
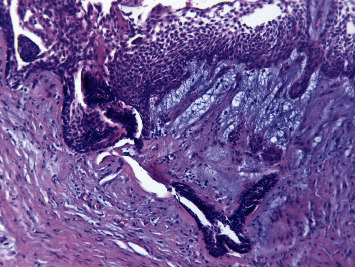
Optical microscopic picture showing the invagination of the ameloblastomatous epithelium inside the fibrous wall with the presence of clusters of ameloblast inside the wall. Stained with hematoxylin and eosin, magnified 60 times.

**Figure 9:**
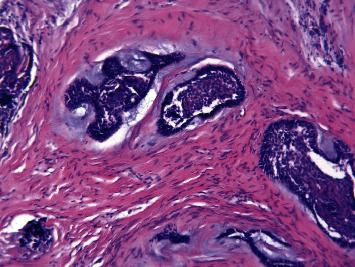
Optical microscopic picture showing infiltrating islands of ameloblast cells presenting a follicular architecture, extending into the connective tissue wall suggestive of the mural variant. Stained with hematoxylin and eosin, magnified 100 times.

The histopathologic evaluation reported a single cystic sac with a fibrous wall and the presence of a lining epithelium showing varying thickness without cytonuclear atypia or mitosis (Figure 7). Inside the wall and the nodule associated with the cyst, ameloblastic epithelial clusters with a follicular architecture were observed without mineral deposit (Figure 8). Foci of ameloblastoma cells were noted in and outside the wall, witnessing extraluminal infiltration (Figure 9). Thus, the diagnosis of mural ameloblastoma subtype was made, contrary to the provisional diagnosis of lateral periodontal cyst.

**Figure 10:**
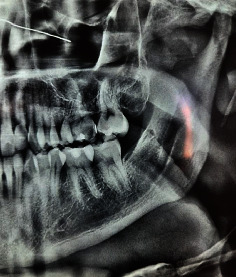
Portion of a 2021 control panoramic radiography focused on the old lesion emplacement.

Two years after the enucleation procedure, the patient is still followed up in the outpatient dental clinic. Bone restoration in the affected area was radiologically observed (Figure 10), and no sign of relapse has been noted to this day.

## 3. Discussion

Unicystic ameloblastoma was first described by Robinson and Martinez in 1977 as one of the three ameloblastoma subtypes: a unique and rare de novo neoplasm. It is reported in 15% of intraosseous ameloblastoma [9]. UA is mostly observed among young patients as noted in our case report. Currently, no gender preference is reported [1, 10]. Eighty percent of the time, UA is reported to be surrounding the crown of impacted teeth [1, 11]. Because of this frequent location, its demarcated unicystic radiolucency, and its low aggressive behavior (compared to conventional ameloblastoma), UA may be mistaken with an odontogenic cyst [12].

Nevertheless, our lesion did not have the usual frequent location reported in the literature (ramus and mandible angle) [13] since it appeared between the 35 and 36 roots. None of these teeth was impacted, and the lesion had no contact with any of their crowns. On the contrary, it was lengthening in continuity with the mesial side of the 35 root and the distal side of the mesial 36 root. Concerning the two remaining root walls (distal side of the 35 root and mesial side of the distal 36 root), periodontal space was no longer radiologically noticeable since the lesion extended a few cm beyond roots' length.

In fact, the location of the lesion, its small radiologically sized aspect, and its intimate contact with the lamina dura made us primarily consider the diagnosis of a lateral periodontal cyst. Some authors reported this same UA differential diagnosis [14, 15], though none of the lesions presented were that small despite years of evolution and had that location. Actually, the intraradicular location is quite rare. Only one article reported a multilocular lesion between the 2nd and 3rd molars, although signs of radicular resorption were observed [13]. Therefore, a biopsy is paramount to elucidate the difference since the culprit lesion has a higher rate of recurrence [16]. In fact, it is only when the pathologist examines the entire specimen that the diagnosis of UA and specifically its subtype can be revealed. In this case presented, considering the small size of the lesion and the differential diagnosis considered, we did not perform a biopsy so the specimen could be removed intact (during the enucleation) and analyzed entirely.

To confirm a UA diagnosis, the minimum criterion is the presence of a single cystic sac lined by variable epithelium ranging from that with typical ameloblastic characteristics to a metaplastic epithelium consisting of nonkeratinizing squamous cell layers [17]. However, it is very important to be aware of the UA histological subtype as it determines, with the procedure, the recurrence rate of the lesion [10, 14].

Ackerman et al. assessed three UA subtypes after a clinicopathologic study of 53 cases [5]:

- The first one is a luminal UA: when the tumor is confined to the luminal surface of the cyst. Its recurrence rate is the lowest, ranging from 10% to 25% since the ameloblastic cells are contained and do not invade adjacent tissue
- The second one is the intraluminal UA: a nodular proliferation into the lumen. No infiltration of the tumor cell into the connective tissue is observed as well. It is usually microscopically similar to conventional ameloblastoma
- The third one is the mural UA with the higher recurrence rate, 50% to 80% risk. Invasive islands of ameloblastomatous epithelium in the connective tissue wall are observed, though they do not involve the entire epithelium. The infiltration can even extend beyond into the surrounding bone, hence its high recurrence rate

Later in 2003, Philipsen and Reichart described another UA grouping as follows [18]:

- Subgroup 1: luminal
- Subgroup 1.2: luminal and intraluminal
- Subgroup 1.2.3: luminal, intraluminal, and intramural
- Subgroup 1.3: luminal and intramural

Treatment—whether conservative or radical—is usually controversial, as it depends on pathology results which may reveal only one of the multiple growth patterns of the lesion [1]. However, the invasive approach is mostly the chosen therapy for the highest recurrence rate groups such as the 1.2.3 and 1.3 subgroups that show intramural growths [8, 10]. Treatment consists of a segmental or marginal resection of the lesion followed by reconstructive plate adjustment or grafting (from the fibula or iliac crest) [19]. It is expected to have the lowest recurrence rate among all treatments: 3.6% if adequate bone margins are removed.

However, opting for this procedure must be well thought out and requires a balanced judgment, so its success does not lead to overtreatment [6, 20]. This option is associated with severe postoperative complications: deformity, oral dysfunctions, etc. [1, 21]. In fact, despite a successful reconstruction; removal of teeth, masticatory dysfunction, and abnormal jaw movement are common and stand against full patient recovery, especially for the fragile [6]. For instance, regarding children, Tanaka et al. demonstrated that minimal surgical treatment must be the first choice [21]. In fact, lack of mandible growth during the developmental period can cause severe facial deformation encroaching deeply on their quality of life [6, 21].

Furthermore, psychological fragility either in children or in adults is put to the test through this severe procedure [22]. Actually, regarding patients with psychological conditions, no specific recommendations were found. It is only reported that in particular cases, positive and negative outcomes must be predicted to find a balanced solution [6, 20].

In our case, our patient had a fragile psychological state as he was suffering from general anxiety disorder (GAD), a chronic disabling psychological disease, for now 10 years [23]. In fact, his condition, including his young age, the small size of the lesion, and the high possibility of a lateral periodontal cyst, was the key point to our decision. Besides, resection procedures and surgery are in general very traumatic, especially for patients with psychological conditions, requiring long and difficult convalescent periods [6]. A study tried to explore the evolution of anxiety of patients who had previous orofacial deformity after an orthognathic surgery [22]. They noticed that even when surgery improved their quality of life and their social connections, it did not change patients' personality, especially regarding anxiety traits. Furthermore, patients might experience significant depression after surgery [22]. In our case, the patient was very disturbed and anxious about his lesion and swelling.

Hence, after comparing the radiological and clinical features of the lesion (its small size, its location between premolar roots, and its continuity with the lamina dura) to the young age and psychological condition of our patient, we opted for a simple enucleation. This procedure would involve less patient morbidity, and the effect on his quality of life is minimal. In fact, literature reported as in our case that some surgeons advocate a conservative approach, especially for young patients to prevent future problems with oral functions and esthetics [1, 24].

Nevertheless, keeping in mind the possibility of an ameloblastoma, we paid attention not to realize a vigorous curettage of the bone as it might implant foci of ameloblastoma more deeply in the bone [25]. A regular follow-up was set with frequent checking appointments. Aside from enucleation, two other conservative procedures exist: marsupialization followed by enucleation and enucleation followed by the application of Carnoy's solution. Enucleation alone has, according to Lau and Sammann, the higher recurrence rate among treatments, 30.5% [20], while the use of Carnoy's solution (suggested by Stoelinga and Bronkhorst in 1988) [26] decreases the risk of recurrence after a conservative surgical treatment. In the present case, we did not use the solution after the enucleation because the lesion destroyed the cortical plate in some portions, becoming in contact with soft tissues and the alveolar vascular nervous plexus. So in this case, the use of Carnoy's solution would have mummified all the anatomical elements surrounding the lesion [27].

Concerning marsupialization, it was not an option either, because of the small size of our lesion, contrary to a 2007 case report presenting a wide lesion in the left region of the mandible managed with a marsupialization [6]. According to a systematic review of Lau and Samman, this procedure has 18% risk of recurrence [20].

Most of the time, conservative treatment is reserved for UA 1 and 1.2 subgroups since no infiltration of ameloblastoma exists beyond the lesion lumen [8]. However, to this day, no real consensus was set concerning UA treatment. No adequate evidence proves which treatment modality is the most effective. Because of the relative rarity of the tumor, a definitive conclusion to this debate is controversial and difficult to reach [6, 20].

In the end, we wanted to give our patient every chance to keep a normal life without encroaching on his already fragile psychology with a difficult procedure and postoperative convalescence. After all, considering the circumstances, the patient is out of danger and satisfied, and we still have 20% to 50% chance of total success [5]. Nevertheless, the patient was informed about the possibility of recurrence considering the true diagnosis of the lesion. After two years, no sign of relapse was observed, although, if recurrence would happen later, a radical procedure should be considered.

## Data Availability

Data sharing is not applicable as no dataset were generated or analyzed in this case report. Images supporting Figures 1–10 are available from the corresponding author on reasonable request.
